# The Emission Characteristics and Health Risks of Firefighter-Accessed Fire: A Review

**DOI:** 10.3390/toxics12100739

**Published:** 2024-10-12

**Authors:** Xuan Tian, Yan Cheng, Shiting Chen, Song Liu, Yanli Wang, Xinyi Niu, Jian Sun

**Affiliations:** 1 School of Human Settlements and Civil Engineering, Xi’an Jiaotong University, Xi’an 710049, China; 3123122029@stu.xjtu.edu.cn (X.T.); cst20020222@163.com (S.C.); m13408425392@163.com (S.L.); yanli2025@stu.xjtu.edu.cn (Y.W.); 2 Department of Occupational and Environmental Health, School of Public Health, Xi’an Jiaotong University Health Science Center, Xi’an 710061, China; 3 Key Laboratory of Environment and Genes Related to Diseases, Ministry of Education, Xi’an 710049, China; 4 Key Laboratory of Trace Elements and Endemic Diseases in Ministry of Health, Xi’an 710049, China; 5 Key Laboratory for Disease Prevention and Control and Health Promotion of Shaanxi Province, Xi’an 710061, China; 6 Department of Environmental Science and Engineering, Xi’an Jiaotong University, Xi’an 710049, China; sunjian0306@mail.xjtu.edu.cn

**Keywords:** air pollution, firefighter, fire emission, health effects

## Abstract

The exacerbation of wildfires caused by global warming poses a significant threat to human health and environmental integrity. This review examines the particulate matter (PM) and gaseous pollutants resulting from fire incidents and their impacts on individual health, with a specific focus on the occupational hazards faced by firefighters. Of particular concern is the release of carbon-containing gases and fine particulate matter (PM_2.5_) from forest fires and urban conflagrations, which exceed the recommended limits and pose severe health risks. Firefighters exposed to these pollutants demonstrate an elevated risk of developing pulmonary and cardiovascular diseases and cancer compared to the general population, indicating an urgent need for enhanced protective measures and health management strategies for firefighters. Through a meticulous analysis of the current research findings, this review delineates future research directions, focusing on the composition and properties of these pollutants, the impacts of fire-emitted pollutants on human health, and the development of novel protective technologies.

## 1. Introduction

Uncontrolled fires have increased in recent decades owing to global warming and the increased frequency of extreme drought. According to data released by China’s National Fire and Rescue Administration, the average daily number of fires in the country during the first half of 2023 exceeded 3000, with a total of 550,000 reported, leading to 959 deaths, 1311 injuries, and CNY 3.94 billion in direct property damage [1]. In the United States, Prestemon et al. predicted that the area burned by wildfires in the southeastern region will further increase by approximately 34% from 2056 to 2060 compared with recent years, with the overall wildfire area expected to grow by 4% [2]. In addition, emissions from fires pose significant risks to both humans and the ecological environment [3,4]. Therefore, it is crucial to raise awareness about the dangers of exposure to fire smoke and implement measures to protect public health during fire incidents.

Fire emissions have a significant impact on the environment. For instance, PM_2.5_ mass concentrations reached a peak of 300 μg m^−3^ during the Sumatra fires, which is 20 times the limit of 15 μg m^−3^ recommended by the World Health Organization (WHO) [5,6]. Griffiths et al. analyzed data from 23 major industrial fires and found that the concentration of PM_10_ was up to 1450 μg m^−3^, while the concentration of PM_2.5_ also reached 258 μg m^−3^ [7]. Some gases from fire incidents, such as carbon dioxide (CO_2_), sulfur dioxide (SO_2_), methane (CH_4_), and carbon monoxide (CO), have emerged as significant contributors to climate warming, air pollution, and heightened acid rain. The release of heavy metals such as mercury and lead poses additional risks to human health, water bodies, soil, and ecosystems [8,9,10].

Firefighters are exposed to large amounts of fire emissions on duty, which pose significant threats to their physical health. Previous health studies have revealed higher incidences of cancer and mortality rates in firefighters compared to the general population [11,12]. However, research on the occupational exposure of firefighters to fire-generated pollutants and the mechanisms underlying the impact of fire emissions on human health remains limited globally.

In this study, we provide a comprehensive overview of fire emissions and their combustion characteristics, as well as the gaseous and particulate pollutants generated. We then outline the health risks associated with various gaseous and particulate pollutants, discuss the potential occupational health risks for firefighters, and elaborate on the pulmonary and cardiovascular diseases associated with fire exposure and their underlying mechanisms. Finally, we summarize existing research findings and outline possible prospects for future academic research in this field.

## 2. Emission Characteristics of Fire

Fire yields immense energy upon ignition, leading to elevated temperatures and the production of copious amounts of smoke and substantial pollutant emissions [13]. Fires can be broadly categorized into forest and urban fires. Urban fires primarily involve building fires, including those occurring in residential or office buildings, factories, shopping malls, vehicles, and waste facilities [14,15,16], which tend to occur near urban areas, resulting in air pollution well above the recommended standards and the serious contamination of soils, water, and agriculture, directly or indirectly, from fire emissions [17]. Forest fires primarily involve the burning of large quantities of vegetation and trees. These two types of emissions differ from each other, with pollutants from urban fires being associated with human-made materials and forest fires emitting pollutants predominantly from biomass burning [18,19]. Urban fires generally produce more toxic and hazardous pollutants compared with other fires [20].

Table 1 summarizes the main pollutants related to fire exposure and their health risks, as outlined in the occupational exposure standards. Among these, the EH40/2005 Workplace Exposure Limits issued by the UK Health and Safety Executive (HSE) specify the maximum allowable concentrations of various harmful substances based on an 8 h Time-Weighted Average (TWA) or a 15-min Short-Term Exposure limit (STEL). Their health impacts were detailed by Reisen et al., who summarized the toxicological patterns of these pollutants. Previous studies have demonstrated that firefighter exposure peaks during fires far exceeding the short-term occupational exposure standards. Miranda et al. monitored the peak CO exposure of firefighters in fires and determined that it reached 421 ppm, far exceeding the standard limit of 100 ppm; their nitrogen dioxide (NO_2_) exposure levels also reached 22 ppm, 22 times the standard of 1 ppm [21]. Barbosa et al. [22] revealed that firefighters’ peak exposure to SO_2_ in fires reached 7600 µg m^−3^, significantly higher than the standard of 2700 µg m^−3^. Thus, these results show that firefighters are exposed to significant cardiovascular, respiratory, and neurological health risks. According to China’s occupational exposure to toxic hazards grading national standard (GB5044-85) [23], benzene and cyanide (-CN) are classified as Class I (extremely hazardous) occupational exposure poisons, with a maximum permissible concentration of 0.1 mg m^−3^. Exposure to these substances may result in acute poisoning, with chronic poisoning prevalence rates exceeding 5%, rendering benzene a confirmed human carcinogen. Formaldehyde, hydrogen fluoride (HF), and similar compounds are classified as Class II (highly hazardous) occupational exposure poisons, with maximum permissible concentrations ranging from 0.1 to 1.0 mg m^−3^. The prevalence of chronic poisoning after human exposure can surpass 20%, and these substances are included in the list of suspected human carcinogens. Dioxins (PCDDs (polychlorinated dibenzo-p-dioxins) and polychlorinated dibenzofurans (PCDFs)), classified as Class I carcinogens by the WHO International Agency for Research on Cancer (IARC) in 1997, possess a stable structure and a long half-life, allowing them to persist in the body following exposure, potentially resulting in serious toxic effects due to prolonged accumulation [24].

### 2.1. Gaseous Pollutants

Carbon-containing gases represent a significant proportion of pollutants emitted from fires, including compounds such as CO, CO_2_, CH_4_, and various non-methane hydrocarbons (NMHCs). This emission profile has significant implications within the framework of China’s dual-carbon target national strategy [30]. Fire incidents frequently involve the combustion of organic matter, which leads to substantial emissions of carbon-containing gases. Zhang et al. revealed that, from 1988 to 2012, the carbon emissions from wildfires in China averaged approximately 1.03 Tg per year, accounting for approximately 0.39% to 0.54% of China’s total carbon emissions [31]. Owing to the increasing frequency of severe wildfires caused by climate change, this proportion may increase in the future. Forest fires produce a significant amount of greenhouse gases, mainly CO_2_, typically accounting for 90% of total carbon emissions [32]. Another study indicated that 63–74% of the carbon emitted from wildfire combustion is in the form of CO_2_, whereas CO and CH_4_ account for approximately 5.7–13% and 0.36–0.53%, respectively [33]. Among carbon-containing gases, CO is a significant contributor to fire casualties. Previous studies have indicated that CO poisoning can lead to myocardial infarction due to potential cardiac arrhythmias, along with other systemic complications such as rhabdomyolysis, renal failure, pancreatitis, and hepatocellular injury [34].

Wildfire emissions are an important source of NMHCs, which play an important role in tropospheric chemistry by promoting the formation of ozone (O_3_) and secondary organic aerosols [35]. NMHCs also make up, along with OVOCs (oxygenated volatile organic compounds), nearly all of the gaseous-phase non-methane organic compounds (NMOCs) emitted by fires, however, there is significant uncertainty regarding the amount of NMOCs emitted from firefields. Akagi et al. measured the unit-area biomass of significant wildfire types and their emissions, and found that wildfire-burning biomass emits at least 400 Tg of gaseous NMOCs annually worldwide, nearly three times higher than previous estimates [36]. Yokelson et al. conducted further studies on the gas emissions from wildfires, and the results showed that Akagi et al.’s results were still an underestimation [37]. Therefore, further research on NMOC emissions from wildfires is necessary. However, limitations in measurement technology, rapid changes in smoke chemistry, the diversity of combustion conditions, and restrictions on the model scale all lead to inaccuracies in quantifying wildfire emissions [37]. This increases the difficulty of research and complicates the assessment of environmental impacts.

Forest fires not only release significant quantities of carbon-containing greenhouse gases, but also emit highly hazardous gases such as formaldehyde (HCHO), acrolein, benzene, hydrogen cyanide (HCN), hydrogen bromide (HBr), SO_2_, and NO_2_ [38,39]. Toxicological studies suggest that these chemicals may activate TRPA1 (a TRP ion channel expressed in chemosensitive C-fibers) through covalent protein modifications, thereby affecting conditions such as asthma and airway inflammation and posing a significant risk of airway injury [40].

### 2.2. Particulate Pollutants

Fires constitute a primary source of global particulate matter pollution that adversely affects air quality and alters the atmospheric composition [41,42], consequently diminishing visibility in the atmospheric environment. PM_2.5_ constitutes over 90% of the total PM emitted from biomass burning [43]. PM_1_ and PM_2.5_ pose significant risks for respiratory diseases and impose a considerable health burden on individuals in their daily lives [44]. Furthermore, the release of various water-soluble inorganic ions from fire emissions can induce soil damage through processes such as acid deposition, thereby exerting profound impacts on ecosystems [45]. Previous studies have shown that, when PM_2.5_ concentrations exceed 50 μg m^−3^, which can occur in burning fields, the decrease in visibility may shift from a linear to an exponential relationship with an increasing PM_2.5_ concentration [46]. Another study demonstrated that the correlation between the PM_10_ concentration and visibility also follows an exponential function [47].

Organic components are the essential chemical constituents of particulate emissions from fire combustion. A previous combustion study showed that particulate-phase polycyclic aromatic hydrocarbons (PAHs) accounted for more than 89% of the total fine particulate matter of combustion smoke [48]. Studies conducted in southeast Asia have highlighted that biomass burning significantly elevates the mass concentrations of fine PM in the region, with the organic component accounting for up to 85% of the total [49]. In a simulated combustion experiment involving various tree types, Alves et al. determined the chemical composition of PM_2.5_, revealing that organic carbon (OC) dominated among the carbonaceous components, accounting for more than 85% of the total carbon (the sum of OC and black carbon [BC]) [50]. BC aerosols are generated by the incomplete combustion of carbon fuels. Water-soluble inorganic ions (e.g., K^+^, Cl^−^, NH_4_^+^, SO_4_^2−^, NO_3_^−^, and NO_2_^−^) can alter the water environment upon entering water bodies, leading to water pollution and affecting water acidity and quality [45]. Additionally, the emission of inorganic ions, such as ammonium (NH_3_), nitrate (NO_3_^−^), sulfate (SO_4_^2−^), and chloride (Cl^−^), in PM emissions can modify atmospheric visibility by influencing the light-scattering properties [51].

### 2.3. Toxic and Hazardous Pollutants from Urban Fires

Urban fires, which occur in diverse settings such as vehicles, waste facilities, shopping malls, factories, and residential buildings, give rise to various types of toxic gases and particles owing to differences in materials, structures, and uses. For instance, Edelman et al. [52] observed a broad spectrum of toxic and hazardous gaseous and particulate pollutants, including PAHs, PCDFs, dibenzodioxins (DDEs), biphenyls (DBPs), cyanide (-CN), and VOCs, in the fire collapse of the World Trade Center. Studies examining combustion emissions from mobile sources in urban areas identified toxic gases such as HCN, HF, hydrochloric acid (HCl), isocyanates (RNCO), PAHs, PCDDs, PCDFs, and NMHCs (benzene, toluene, xylene, styrene, and formaldehyde) [53]. Among organic compounds, PAHs have the strongest toxicity and pose significant health risks, making them of concern to the public.

Combustion emissions are the major source of these harmful PAHs [54]. PAHs, which are commonly produced as byproducts of incomplete combustion, have been widely demonstrated to be toxic. Seven PAH compounds, namely benzo(a)anthracene, benzo(a)pyrene, benzo(b)fluoranthene, benzo(k)fluoranthene, toluene, dibenzo(ah)anthracene, and benzo(ghi)perylene, have been identified as potential human carcinogens. In a large-scale fire experiment, researchers tested a variety of items, including televisions, electrical cables, furniture, rubber tires, and electronic waste, to simulate common urban fire scenarios. The main PAHs released during these experiments included naphthalene, acenaphthalene, acenaphthene, fluorene, phenanthrene, and anthracene, as well as the more toxic benzo[a]anthracene, chrysene, benzo[b]fluoranthene, and benzo[k]fluoranthene [55]. Benzo [a]pyrene is a widely studied PAH known for its carcinogenic metabolites. High-molecular-weight PAHs such as indeno [1,2,3-cd]pyrene and benzo[ghi]perylene, although present at lower concentrations, exhibited significant toxicity. Dibenz[a,h]anthracene is highly toxic and poses a particular danger to the environment. These studies not only revealed the characteristics of PAH emissions during the combustion of various materials, but also assessed the potential health and environmental risks associated with these compounds, highlighting their importance for understanding the environmental impacts post-fire and enhancing public safety.

PAHs may induce cardiovascular and respiratory diseases and cancer [56]. In the short term, PAHs can cause skin irritation and inflammation [57]. Compounds such as anthracene, benzo(a)pyrene, and naphthalene are direct skin irritants, whereas anthracene and benzo(a)pyrene are skin sensitizers that cause allergic skin reactions in animals and humans [58]. Long-term exposure to PAHs poses health threats primarily in terms of teratogenicity and carcinogenicity. Laboratory studies have shown that animals exposed to long-term PAHs have an increased risk of lung, gastric, and skin cancers [56]. PAHs can also affect the hematopoietic and immune systems, causing reproductive, neurological, and developmental effects [59]. Benzo(a)pyrene, one of the first identified carcinogenic PAHs, has been demonstrated in mouse experiments to reduce offspring weight with exposure to high concentrations during pregnancy [60]. Additionally, research suggests that prenatal exposure to high concentrations of PAHs may impact children’s intelligence [61]. Firefighters face higher levels of PAH exposure during firefighting activities, thereby encountering elevated risks associated with PAH exposure [62].

### 2.4. Impact of Combustion Condition on Pollutant Emissions

Pollutant emissions during fires are closely linked to the combustion stages, typically classified as smoldering and flaming, which often occur simultaneously [63]. This distinction can be quantified using modified combustion efficiency (MCE), which can be estimated as Δ [CO_2_]/(Δ [CO_2_] + Δ[CO]), where Δ [CO_2_] and Δ[CO] are the molar amounts of CO_2_ and CO, respectively. This reflects the relative amount of combustion between flaming and smoldering [64]. An MCE of 0.9–1.0 predominantly signifies flaming, whereas an MCE less than 0.9 indicates smoldering [65]. The primary factors influencing flaming and smoldering are temperature, size, shape, fuel density, and moisture content [66,67]. Generally, a smaller fuel size, lower moisture content, and looser packed fuels facilitate flaming, whereas smoldering tends to increase with a higher fuel moisture content [68]. It is important to note that the pollutants produced by flaming and smoldering exhibit significant differences in composition and size. For instance, flaming combustion typically yields CO_2_, NO_x_, HCl, SO_2_, nitrites (HONO), and BC, whereas smoldering combustion is primarily associated with CO, CH_4_, NH_3_, and various HCs and organic aerosols (OAs) [69,70]. Carrico et al. [71] demonstrated that the particulate matter produced by smoldering (MCE < 0.95) is dominated by particles larger than 100 nm, whereas flaming (MCE > 0.95) produces particles smaller than 50 nm. Additionally, the PM_2.5_ emission factor of smoldering is higher than that of flaming, with smoldering emitting higher concentrations of organic matter such as levoglucan, oxalate, acetic acid, and formic acid, while flaming emits proportionally greater amounts of inorganic fractions [72].

## 3. Health Risks of Firefighters’ Occupational Exposure

**Figure 1:**
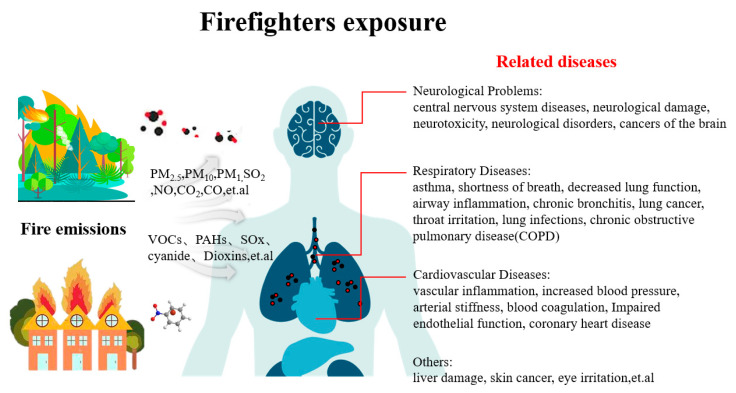
Occupational exposure of firefighters [73,74,75,76,77,83,84,85,86,87,88,89,90].

Throughout their careers, firefighters are exposed to large amounts of toxic and hazardous pollutants during firefighting and rescue operations. Continuous exposure to toxic gases and particulate matter can cause irreversible damage to the respiratory and cardiovascular systems, as well as overall health [62]. Numerous environmental health studies have demonstrated the health risks posed by fine particulate matter and various gaseous and particulate combustion fractions, which encompass a wide range of neurological, respiratory, and cardiovascular disorders [73,74,75,76,77]. Figure 1 illustrates the health problems to which firefighters are exposed. It briefly summarizes the contaminants to which firefighters are exposed to during a fire and the health risks that they may face. The potential occupational health risks to firefighters may be underestimated because of the limited amount of research on firefighters’ occupational health exposure, most of which is limited to lung function and cardiovascular diseases.

We have also noticed that the health of firefighters, a high-risk profession, is impacted by personal protective equipment (PPE, including firefighting vests, firefighting suits, and particle-blocking hoods). Despite most countries equipping firefighters with PPE, many studies indicate that this equipment cannot provide adequate protection. Mayer et al. suggested that fire emissions can penetrate firefighting suits, including volatile toxic gases like benzene and naphthalene, as well as particulate-bound polycyclic aromatic hydro-carbons (PAHs). These pollutants may bypass or penetrate the protective barriers of fire-fighting suits and hoods, posing threats to firefighters’ skin and respiratory systems [78,79]. Self-contained breathing apparatuses (SCBAs) and protective hoods are generally considered to be crucial in preventing exposure to particulates and toxic gases. However, Fent et al. indicated that, even with SCBAs, the benzene levels in exhaled breath increase after firefighting tasks [80]. The protective performance of hoods decreases with repeated use [81]. A study has shown that the absorption of toxic pollutants by the body increases with skin temperature. Since firefighters often work in high-temperature environments, their health risks may be further exacerbated [82]. To address the health risks faced by firefighters, many countries conduct regular health monitoring and exposure assessments. They also schedule work rotations reasonably and provide scientifically based nutritional guidance for firefighters. However, recent research on firefighters’ health suggests that these strategies may be insufficient to protect their health. The following is a systematic review of selected articles on occupational exposure among firefighters.

### 3.1. Healthy Risks of Lung Diseases

Some studies have analyzed the health risks of firefighting in humans by monitoring lung function before and after fires [83,91,92,93]. Several hours of exposure to these particles may lead to reduced ciliary activity in the respiratory tract, compromising the body’s ability to fend off respiratory infections [94]. Previous studies have typically used pulmonary function indices to assess lung health and function, including forced vital capacity (FVC), forced expiratory volume in one second (FEV_1_), park expiratory flow (PEF), and forced expiratory volume in one second percentage (FEV_1_% rate). Jacquin et al. [95] found significant decreases in lung function indices among French firefighters early in the wildfire season following exposure to coniferous smoke. Similar observations were made among firefighters in the United States, as well as an increase in cases of seasonal chronic bronchitis [84]. Studies have highlighted the detrimental effects of high smoke concentrations on firefighters’ lung function. Moreover, the sustained occupational exposure of firefighters to smoke may exert a cumulative effect on lung function. In the southeastern United States, firefighters exhibited a decreasing trend in lung function indices with each additional day of work at prescribed burn sites during the winter months. Specifically, their FVC and FEV_1_ decreased by 24 mL [85]. This cumulative effect persisted for one or two burn seasons [84]. Gaughan et al. [86] observed an association among exposure, oxidative stress, symptoms, and cardiorespiratory function in wildland firefighters. They suggested that decreased lung function may be linked to the smoke tracer L-glucan produced during burning.

Firefighters may encounter an increased risk of cancer due to exposure to various particulate matter and noxious gases. Studies have typically measured different pollutant concentrations (e.g., PM_4_ and PM_2.5_) and respiration rates in various firefighting scenarios [92,96,97]. The daily intake of PM_2.5_ was estimated using measured pollutant concentrations, firefighter respiration rates, daily working hours, and frequency of exposure. Subsequently, the relative risk of lung cancer mortality was evaluated based on the exposure–response relationships associated with PM_2.5_ [92,96]. The exposure–response relationship describes the association between exposure dose and health effects with scattering plots of the estimated average daily dose of pollutants and mortality. This relationship can be linear or nonlinear, depending on how changes in exposure levels affect the extent and trend of health effects [97]. For instance, during the most intense fires in Tuolumne County, people could intake an average of 0.49 mg of PM_2.5_ per day, with a maximum of 1.31 mg, and researchers found that the daily dose of PM_4_ exposure was linearly related to health risks, posing a clear threat to lung health [92]. Furthermore, this elevated risk may be attributed to exposure to toxic substances such as benzene and formaldehyde during firefighters’ work [98]. Daniels et al. [99,100] noted a linear relationship between lung cancer and cumulative exposure (e.g., to PAHs, HCHO, benzene, 1,3-butadiene, asbestos, and arsenic) and found that lung cancer mortality was positively correlated with the duration of exposure.

### 3.2. Health Risks of Cardiovascular Diseases

Coronary heart disease has emerged as the leading cause of line-of-duty deaths among U.S. firefighters, with approximately 45% of all fatalities annually attributed to sudden cardiac events [101]. Air pollution is associated with myocardial ischemia and cardiac diseases [87,88]. The case-crossover approach, which integrates data on pollutants and data on patients with cardiovascular disease, has been widely used to identify the triggering factors for the onset of cardiovascular events [89]. Dockery et al. demonstrated a significant increase in heart rate with elevated PM_10_ levels, indicating a heightened cardiovascular risk due to exposure to air pollution [102]. Exposure to chemical asphyxiants at fire scenes, such as CO, cyanide (-CN), and hydrogen sulfide (H_2_S), interferes with oxygen transport and cellular respiration, leading to tissue hypoxia and subsequent myocardial ischemia in individuals with underlying cardiovascular conditions [90]. Previous studies on exposure–response relationships showed close correlations between pollutants (PM_2.5_, PM_4_, PAHs, HCHO, benzene, 1,3-butadiene, asbestos, and arsenic) and cardiovascular disease mortality [92,96,99,100].

Related studies have shown that firefighting, as a strenuous and dangerous occupation, poses a higher relative risk of cardiovascular disease mortality [103,104]. Previous studies have shown that the proportion of fatal cardiovascular events among firefighters significantly surpasses that of other high-intensity manual labor occupations, such as construction workers (12%) and police officers (22%) [105,106,107]. Fire suppression duties, characterized by strenuous physical exertion, pose a particularly high risk of sudden cardiac death and associated cardiovascular ailments [103]. Other factors contributing to the elevated prevalence of cardiovascular disease among firefighters include obesity, hypertension, smoking, and dyslipidemia [108]. Firefighters’ habits (e.g., smoking, exercise, and diet smoke) affect obesity, which also increases the risk of cardiovascular disease, suggesting that lifestyle factors outside the occupation may exacerbate these health risks [109,110].

Thus, firefighters face substantial risks of cardiovascular diseases due to occupational exposure and lifestyle factors. Despite potential differences in diet, exercise habits, and health awareness across regions, the threat posed by smoke exposure to cardiovascular health remains significant.

### 3.3. Other Health Risks

The risks faced by firefighters extend beyond pulmonary and cardiovascular diseases to include an elevated risk of various types of cancer. Scholars in the medical field have utilized meta-analyses to investigate the cancer risk among firefighters, consistently finding that firefighters are at a heightened risk of cancer [111,112,113].

In July 2022, the WHO reported that firefighters are exposed to fire combustion products containing contaminants such as PAHs, VOCs, particulate matter, and persistent organic compounds present in firefighting foams. Much of this mixture of exposures has been classified by the IARC as Groups I (carcinogenic to humans), 2A (probably carcinogenic to humans), and 2B (possibly carcinogenic to humans). Relevant studies have supported these findings. For instance, in a study on the mortality and cancer incidence among male firefighters in Australia, Glass et al. observed an increased risk of cancer among these male firefighters [114]. Similar results were confirmed in a Norwegian study that analyzed data from Norwegian firefighters spanning nearly 50 years, revealing elevated rates of colon cancer, mesothelioma, and prostate cancer compared with the general population [115]. Furthermore, other studies have highlighted the heightened risks of cancers of the digestive system, testicular cancer, and various other cancers in firefighters [100,116]. These findings highlight the increased cancer risk associated with firefighters’ occupational exposure, underscoring the need for a better understanding of the contaminants in their work environments and the development of more effective occupational safety measures and health safeguards for firefighters.

## 4. Studies on the Pathogenic Mechanisms of Fire Emissions

Recent studies have used both in vitro (cell exposure) and in vivo (mouse exposure) experiments to explore the pathogenic mechanisms of cardiovascular disease and lung injury caused by pollutant exposure. Particulate matter, particularly PM_2.5_, is closely associated with respiratory and cardiovascular diseases [117,118] and has been extensively utilized in both cell exposure and mouse exposure experiments. Toxicological experimental studies focusing on particulate matter have increased in recent years, yielding valuable insights into the effects of particulate matter on health outcomes.

### 4.1. In Vitro Experiments

Cellular experiments involve biological investigations conducted on living cells to observe their cellular responses under various conditions. In particulate exposure experiments, typical procedures include cell pretreatment, cell culture, and cytotoxicity testing, where cytotoxicity denotes the extent of cell damage resulting from substance stimulation. These experiments facilitate the elucidation of cellular stress response mechanisms to diverse stimuli and environmental conditions and have seen increasing utilization in personal exposure studies over time [119,120]. Prolonged exposure to toxic and hazardous environments poses a significant threat to firefighters’ cardiovascular, pulmonary, and respiratory systems.

Human lung epithelial cells (A549 cells) [119,120,121,122,123] and bronchial epithelial cell lines (BEAS-2B) [124,125] are commonly employed in studies involving damaged bronchial or lung cells. Vascular smooth muscle cells (VSMCs) are frequently used to investigate cardiovascular injuries [126]. The A549 cell line, derived from human alveolar adenocarcinoma tissues, possesses characteristics similar to those of lung cancer cells, making it valuable for studying lung cancer pathogenesis. The BEAS-2B cell line, which represents human bronchial epithelial cells, is widely used for simulating airway diseases, toxicant exposure, and cytotoxicity. Commonly assessed cytotoxicity indicators include reactive oxygen species (ROS), malondialdehyde (MDA), Interleukin-6 (IL-6), Monocyte Chemoattractant Protein-1 (MCP-1), and 8-Hydroxy-2′-deoxyguanosine (8-OHdG), which reflect oxidative stress, membrane lipid peroxidation, inflammatory response, and DNA damage, respectively [127]. Monitoring the levels of these indicators provides insights into the extent of oxidative or inflammatory responses induced by particulate matter exposure, facilitating a comprehensive understanding of adverse health effects at the cellular level.

Ahmed et al. [128] conducted a study on the toxicological effects of various PM sources on BEAS-2B cells and revealed significant genotoxic and mutagenic effects. Their findings highlighted that the PAHs within PM trigger an elevation in ROS levels, leading to oxidative stress and subsequent damage to biomolecules such as DNA, lipids, and proteins, contributing to the development of diseases such as cancer [129]. Additionally, exposure to PM enhances VSCM migration, increases intracellular ROS levels, and disrupts mitochondrial function, potentially leading to vasculopathy and exacerbating cardiovascular diseases over time [130]. Sun et al. [131] noted, in their investigation into the oxidative-stress-induced effects of PM_2.5_ on human lung epithelial cells in ten major cities in northern China, that PM_2.5_ road dust led to significantly higher levels of ROS in A549 cells compared to the control group. High levels of ROS are also a major contributor to the cytotoxicity associated with PM_2.5_ emissions from solid fuels [132]. Furthermore, Dergham et al. [124] noted in their research that oxidative damage and inflammatory responses preceded cytotoxicity in BEAS-2B cells exposed to PM_2.5–0.3_ air pollution. They highlighted the role of inorganic components as exogenous inducers of oxidative damage and the inflammatory response. Although cellular experiments offer advantages such as controllable experimental conditions, cost effectiveness, and ease of operation, they also have limitations. These experiments can only explore specific cell types or organs and cannot fully replicate the complex physiological and metabolic responses of living organisms. Moreover, the concentrations of the substances used in these experiments may exceed physiological levels, limiting their ability to mimic real-life conditions and provide a comprehensive assessment.

Cellular experiments offer a pathway to achieving a deeper comprehension of the health hazards confronting firefighters during occupational exposure, creating a scientific framework for the formulation of more efficacious health management and protective strategies. Nonetheless, the majority of pollutants utilized in prior cellular exposure experiments in China originated from atmospheric sources [133,134], highlighting the lack of research on cellular experiments concerning fire-related pollutants.

### 4.2. In Vivo Experiment (Mouse Exposure)

In recent years, mouse experiments have emerged as a pivotal tool in toxicological research, particularly for investigating the hazards posed by atmospheric particulate matter to human health [135,136]. These experiments investigate the potential health risks associated with particulate matter by subjecting mice to controlled environmental pollutant conditions and meticulously scrutinizing the impacts of particulate matter on their physiological systems, inflammatory responses, gene expression, and other pertinent toxicological indicators. Mouse experiments typically fall into two categories, short- and long-term exposure studies, which are distinguished primarily by the duration of exposure, frequency of testing, concentrations of the pollutants administered, and assessment of outcomes. Short-term exposure typically spans a brief period, ranging from a few hours to several days, and involves higher doses, with a focus on acute effects such as abrupt alterations in physiological markers. Conversely, long-term exposure experiments extend from weeks to months, employ lower doses, and concentrate on chronic toxic effects.

Mouse exposure studies are commonly used to study short-term pollution exposure. Jiang et al. [137] conducted an experiment on mice to study the short-term effects of pollution exposure. Mice were randomly divided into five groups of eight and injected with different doses of particulate matter, including a low-dose group (1.6 mg/kg BW), medium-dose group (8.0 mg/kg BW), and high-dose group (40.0 mg/kg BW), as well as a physiological saline control group and a blank control group. Lactate dehydrogenase (LDH), alkaline phosphatase (AKP), and acid phosphatase (ACP) levels and the activities of cytokines such as tumor necrosis factor-alpha (TNF-alpha) and interleukin 1 (IL-1) in the bronchoalveolar lavage fluid were determined. The results showed that the inhalation of PM_2.5_ induced a collective immune response and oxidative stress, resulting in toxic effects on the mouse lung parenchymal cells and membranous tissues. Moreover, PM_2.5_ triggered oxidative-stress-dependent inflammation in regions rich in type II alveolar cells in the lungs [138]. Oxidative stress may be a causative factor of chronic inflammation, potentially leading to emphysematous chronic obstructive pulmonary disease [138]. A two-week mouse experiment highlighted that the synergistic effect of formaldehyde and PM_2.5_ induced oxidative damage and apoptosis in mouse lung tissues [139]. Animal experiments have contributed significantly to the research on cardiovascular diseases. One study conducted intratracheal instillation to administer varying doses of PM_2.5_ into the lungs of rats, and assessments were conducted on their serum myocardial enzymes (LDH and creatine kinase isoenzyme (CK-MB)), blood pressure, heart rate, and electrocardiogram post-exposure to the pollutant [140]. They observed that PM_2.5_ had a toxic effect on the cardiovascular system in rats, positively correlating with serum LDH and CK-MB creatine kinase isoenzyme levels, indicative of cardiovascular damage [140]. Additionally, Zhang et al. [141] conducted exposure experiments on combustion-generated SO_2_, NO_2_, and PM_2.5_, revealing myocardial mitochondrial abnormalities, inflammatory cell infiltration, and endothelial dysfunction, which could culminate in inflammation and subsequent heart disease.

Mouse exposure studies are not only widely utilized in short-term exposure assessments, but also hold significant value in long-term investigations. A study conducted pathological observations of the hearts and lungs of mice 28, 56, and 84 days after a tail vein injection of a PM_2.5_ suspension. The results showed that prolonged exposure to PM_2.5_ resulted in severe damage, such as endothelial defects and the detachment of blood vessels [142]. Yang et al. [143] placed mice in different locations with high and low concentrations of PM_2.5_. After three months, obvious tissue damage was observed in the contaminated group, marked by increased numbers of inflammatory cells, neutrophils, polylymphocytes, and eosinophils compared with the control group. In addition, the levels of IL-4 (Interleukin-4), TNF-α (Tumor Necrosis Factor α), and TGF-β1 (Transforming Growth Factor-β) were significantly elevated in both the serum and tissues of the polluted group, suggesting that PM_2.5_ pollutants can induce lung injury and significantly influence the inflammatory cytokine levels in mice. Prolonged exposure to high levels of PM_2.5_ often significantly increases the basal blood pressure, accompanied by an increased low-frequency blood pressure variability and urinary norepinephrine excretion, all of which increase the risk of cardiovascular disease [144].

However, in Tuolumne County, the average daily intake of PM_2.5_ averaged 0.49 mg, with the highest reaching 1.31 mg per day [92]. Firefighters exposed to fires for longer durations may have even higher intake levels. In Jiang et al. [137], the low, medium, and high concentrations that the mice were exposed to were 1.6, 8, and 40 mg/kg, respectively. Higher experimental doses may help to clarify the potential health impacts of shorter exposure periods and allow for the simulation and study of worst-case scenarios. This approach will help to better prepare and protect firefighters from potential long-term health effects. Therefore, mouse experiments are important for understanding the health implications faced by firefighters. Despite the insights provided by mouse exposure experiments on particulate matter, the pathogenic mechanisms exerted by fire pollutants on firefighters’ occupational exposure and human health remain relatively understudied and warrant further refinement, owing to the complexity of fire pollutant composition and the specificity of firefighter occupations.

## 5. Knowledge Gaps and Research Opportunities

In this study, we detailed the pollution emissions from fires and the occupational risks faced by firefighters and demonstrated their harm to the respiratory and cardiovascular systems through cellular and mouse experiments. Although our study provides a basic understanding of these effects, there are still several key knowledge gaps that offer significant opportunities for future research.

Despite evidence indicating a link between fire smoke and various respiratory and cardiovascular diseases, the specific pathogenic mechanisms and long-term effects of this remain unclear, and sometimes, there are even inconsistencies. A study in North Carolina showed that emergency department visits for congestive heart failure were associated with exposure to fire smoke [145], yet Johnston et al. did not find an association between wildfire smoke and emergency department cardiac failures [146]. The scarcity of research and these inconsistencies have contributed to a vague understanding of the impacts of fires on cardiovascular and other diseases. Future research should extensively discuss the relationship between fire exposure and specific cardiovascular diseases and explore how these exposures affect health at the biomolecular level through toxicology.

Another shortcoming is the lack of research on the factors affecting wildfire smoke and corresponding response strategies. Current studies have already demonstrated the significant health risks posed by fires, and research has introduced a community health vulnerability index to identify communities susceptible to fire impacts, thereby providing effective guidance for public health infrastructure [147]. However, the high population density in Chinese cities significantly amplifies the impact of urban fires, for which there is a notable lack of global research. Consequently, there is a pressing need for new models and tools specifically designed to assess the risks associated with urban fires, which would facilitate more effective smoke control and prevention strategies.

Furthermore, given the extensive damage and high risks associated with fires, affected populations exhibit a variety of occupations and characteristics, leading to differing levels of susceptibility. Research has identified high-risk groups that are more susceptible to the effects of fire, including individuals with respiratory conditions, potential cardiovascular diseases, people over 65, children, pregnant women, and fetuses [148]. With the global trend of aging populations and the increasing prevalence of respiratory and cardiovascular diseases post-COVID-19, more people are at risk from fire smoke. Therefore, there is a need for further research on wildfire exposure assessments, as well as susceptible populations and professions. More accurate smoke prediction models, additional follow-up studies, and toxicological research are required to better understand and mitigate these risks.

## 6. Conclusions

Our research reveals the extensive impact of fire smoke on human health, particularly among occupational groups such as firefighters. However, our findings highlight many key knowledge gaps, especially regarding the specific pathogenic mechanisms of wildfire smoke, its long-term health impacts, and effective protective measures. Moreover, owing to the inconsistencies and limitations of this research, future studies need to consider the susceptibility of different populations more comprehensively, develop more accurate tools for assessing smoke exposure, and utilize a multidisciplinary approach combining epidemiology, toxicology, and model predictions to further investigate the specific impacts of fire smoke on human health. This will provide a scientific basis for developing more effective public health strategies and help to protect those most vulnerable to these impacts, ensuring their health and safety.

## Figures and Tables

**Table 1:** Occupational standards of typical hazardous pollutants and their health effects.

	Standard Level		Standard	Health Effects	Ref.
Pollutant	Long-Term Exposure	Short-Term Exposure			
Formaldehyde	2 ppm/2.5 mg m^−3^	2 ppm/2.5 mg m^−3^	EH40/2005 Workplace exposure limits	Causes cancer and/or heritable genetic damage.	[25,26]
CO	20 ppm/23 mg m^−3^	100 ppm/117 mg m^−3^	EH40/2005 Workplace exposure limits	Leads to heart disease and damage to the nervous system and causes headaches, dizziness, and fatigue.	[26,27]
NO_2_	0.5 ppm/0.96 mg m^−3^	1 ppm/1.91 mg m^−3^	EH40/2005 Workplace exposure limits	Damage to liver, lungs, spleen, and blood. Can aggravate lung diseases, leading to respiratory symptoms and increased susceptibility to respiratory infection.	[26,27]
SO_2_	0.5 ppm/1300 µg m^−3^	1 ppm/2700 µg m^−3^	EH40/2005 Workplace exposure limits	Aggravates asthma, reduces lung function, and inflames the respiratory tract. Causes headaches, general discomfort, and anxiety.	[26,27]
Benzene	1 ppm/3.25 mg m^−3^	n.a	EH40/2005 Workplace exposure limits	A human carcinogen which can cause leukemia and birth defects. Can affect the central nervous system and normal blood production, and can harm the immune system.	[26,27]
Cyanide ^a^	5 mg m^−3^	n.a	EH40/2005 Workplace exposure limits	Human carcinogen.	[23,26]
HF	1.8 ppm/1.5 mg m^−3^	3 ppm/2.5 mg m^−3^	EH40/2005 Workplace exposure limits	Suspected human causative agent.	[23,26]
VOCs ^b^	n.a	n.a	International Agency for Research on Cancer ^c^	Respiratory toxicity, neurovascular toxicity, carcinogenicity, and liver and kidney toxicity	[23,28]
PM_2.5_	5 µg m^−3^	15 µg m^−3^	WHO	Causes or aggravates cardiovascular and lung diseases, heart attacks, and arrhythmias, affects the central nervous system and the reproductive system, and causes cancer. The outcome can be premature death.	[27,29]

n.a: not recorded in the standards; ^a^: except HCN, cyanogen, and cyanogen chloride (as Cn); ^b^: Volatile organic compounds; ^c^: The IARC has classified carcinogens, including some VOCs, such as formaldehyde, benzene, 1,3-butadiene, and dioxins as Group 1 carcinogens (carcinogenic to humans), while dichloromethane and benz[a]anthracene are classified as Group 2A carcinogens (probably carcinogenic to humans).

## Data Availability

No data were used for the research described in the article.
